# Supplementary material for: Changes in COVID-19 Vaccine Acceptability among Parents with Children Aged 6–35 Months in China—Repeated Cross-Sectional Surveys in 2020 and 2021
Source: Vaccines (Basel). 2023 Jan 12;11(1):170. doi: 10.3390/vaccines11010170 (PMC9867369; doi:10.3390/vaccines11010170)
Supplement: Supplementary file 1 [file vaccines-11-00170-s001.zip › vaccines-2133622-supplementary.docx]

**Material S1**

**Questionnaire used in the first round of survey**

**Part 1:**

1-1 Do you have any child?

□_1_ No □_2_ Yes, all of them are at least 18 years old

□_3_ Yes, at least one of them is under the age of 18 years --- Please answer the following questions

1-2 How old is your child who is under the age of 18 years? (If you have more than one child under the age the age of 18 years, please refer to the one whose birthday is closest to today when answering the following questions)

【 】years

1-3 COVID-19 vaccines developed by China are likely to become available by the end of 2020. What is your likelihood of having your child under the age of 18 years take up free COVID-19 vaccination provided by the government?

□_1_ Very unlikely □_2_ Unlikely □_3_ Neutral □_4_ Likely □_5_ Very likely

**Part 2:**

2-1 Do you agree with the following statements related to COVID-19 vaccination? There is no right or wrong answers.

|  | **Disagree** | **Neutral** | **Agree** |
| --- | --- | --- | --- |
| A. COVID-19 vaccination is highly effective in protecting your child from COVID-19 | 1 | 2 | 3 |
| B. Taking up COVID-19 vaccination can contribute to the control of COVID-19 in China | 1 | 2 | 3 |
| C. China will have adequate supply of COVID-19 vaccination | 1 | 2 | 3 |
| D. Your child will have severe side effects after receiving COVID-19 vaccination | 1 | 2 | 3 |
| E. The protection of COVID-19 vaccines will only last for a short time | 1 | 2 | 3 |
| F. Your child is afraid of vaccination | 1 | 2 | 3 |
| G. You do not have time to take your child for COVID-19 vaccination | 1 | 2 | 3 |
| H. Your family member would support you in having your child take up COVID-19 vaccination | 1 | 2 | 3 |
| I. Having the child receive COVID-19 vaccination is easy for you if you want them to | 1 | 2 | 3 |

2-2 Frequency of exposing to the following information related to COVID-19 vaccination on social media (WeChat, WeChat moments, Weibo, Tiktok, etc.) in the past month

|  | **Almost never** | **Seldom** | **Sometimes** | **Always** |
| --- | --- | --- | --- | --- |
| A. Experiences related to COVID-19 vaccination shared by recipients | 0 | 1 | 2 | 3 |

2-3 Frequency of facemask wearing in public places/transportations other than workplaces in the past month

□_1_ Every time □_2_ Often □_3_ Sometimes □_4_ Never

2-4 Frequency of facemask wearing when you have close contact with other people in workplace in the past month

□_1_ Every time □_2_ Often □_3_ Sometimes □_4_ Never

2-5 Frequency of sanitizing hands (using soaps, liquid soaps or alcohol-based sanitizer) after returning from public spaces or touching public installation

□_1_ Every time □_2_ Often □_3_ Sometimes □_4_ Never

| 2-6 In the past month, did you…… | **Yes** | **No** |
| --- | --- | --- |
| A. Avoid social/meal gathering with other people who do not live together | 1 | 2 |
| B. Avoid crowed places | 1 | 2 |

**Last part:**

3-1 How old are you: _____ years

3-2 What is your gender? □_1_Male □_2_ Female

3-3 What is your relationship status?

□_1_ Without a stable boyfriend/girlfriend

□_2_ With a stable boyfriend/girlfriend

□_3_ Married

□_4_ Divorced/widowed

3-4 What is your education level?

□_1_ Primary school or below

□_2_ Junior high

□_3_ Senior high or equivalent

□_4_ College

□_5_ University

□_6_ Postgraduate

3-5 What is your monthly income level?

□_1_ Below 1000RMB

□_2_ 1000-2999 RMB

□_3_ 3000-4999 RMB

□_4_ 5000-6999 RMB

□_5_ 7000-9999 RMB

□_6_ 10,000 RMB or above

□_7_ No fixed income

3-8 Are you a frontline worker or a management staff?

□_1_ Frontline worker □_2_ Management staff

**End of Questionnaire**

**Questionnaire used in the second round of survey**

**Part 1:**

1-1 Do you have any child?

□_1_ No □_2_ Yes, all of them are at least 18 years old

□_3_ Yes, at least one of them is under the age of 18 years --- Please answer the following questions

1-2 How old is your child who is under the age of 18 years? (If you have more than one child under the age the age of 18 years, please refer to the one whose birthday is closest to today when answering the following questions)

【 】years

1-3 Number of doses of COVID-19 vaccination received by your child

□_1_ 0 --- please answer 1-3A □_2_  1 □_3_ 2

1-3A What is your likelihood of having your child under the age of 18 years take up free COVID-19 vaccination provided by the government?

□_1_ Very unlikely □_2_ Unlikely □_3_ Neutral □_4_ Likely □_5_ Very likely

**Part 2:**

2-1 Do you agree with the following statements related to COVID-19 vaccination? There is no right or wrong answers.

|  | **Disagree** | **Neutral** | **Agree** |
| --- | --- | --- | --- |
| A. COVID-19 vaccination is highly effective in protecting your child from COVID-19 | 1 | 2 | 3 |
| B. Taking up COVID-19 vaccination can contribute to the control of COVID-19 in China | 1 | 2 | 3 |
| C. China will have adequate supply of COVID-19 vaccination | 1 | 2 | 3 |
| D. Your child will have severe side effects after receiving COVID-19 vaccination | 1 | 2 | 3 |
| E. The protection of COVID-19 vaccines will only last for a short time | 1 | 2 | 3 |
| F. Your child is afraid of vaccination | 1 | 2 | 3 |
| G. You do not have time to take your child for COVID-19 vaccination | 1 | 2 | 3 |
| H. Your family member would support you in having your child take up COVID-19 vaccination | 1 | 2 | 3 |
| I. Having the child receive COVID-19 vaccination is easy for you if you want them to | 1 | 2 | 3 |

2-2 Frequency of exposing to the following information related to COVID-19 vaccination on social media (WeChat, WeChat moments, Weibo, Tiktok, etc.) in the past month

|  | **Almost never** | **Seldom** | **Sometimes** | **Always** |
| --- | --- | --- | --- | --- |
| A. Experiences related to COVID-19 vaccination shared by recipients | 0 | 1 | 2 | 3 |
| B. COVID-19 pandemic is not under control in some countries after scaling up COVID-19 vaccination | 0 | 1 | 2 | 3 |
| C. Infectiousness and harms of the variants concern of COVID-19 | 0 | 1 | 2 | 3 |
| D. Outbreak caused by variants concern of COVID-19 in some places of China | 0 | 1 | 2 | 3 |
| E. People contract COVID-19 after receiving primary series of COVID-19 | 0 | 1 | 2 | 3 |

2-3 Frequency of facemask wearing in public places/transportations other than workplaces in the past month

□_1_ Every time □_2_ Often □_3_ Sometimes □_4_ Never

2-4 Frequency of facemask wearing when you have close contact with other people in workplace in the past month

□_1_ Every time □_2_ Often □_3_ Sometimes □_4_ Never

2-5 Frequency of sanitizing hands (using soaps, liquid soaps or alcohol-based sanitizer) after returning from public spaces or touching public installation

□_1_ Every time □_2_ Often □_3_ Sometimes □_4_ Never

| 2-6 In the past month, did you…… | **Yes** | **No** |
| --- | --- | --- |
| A. Avoid social/meal gathering with other people who do not live together | 1 | 2 |
| B. Avoid crowed places | 1 | 2 |

**Last part:**

3-1 How old are you: _____ years

3-2 What is your gender? □_1_Male □_2_ Female

3-3 What is your relationship status?

□_1_ Without a stable boyfriend/girlfriend

□_2_ With a stable boyfriend/girlfriend

□_3_ Married

□_4_ Divorced/widowed

3-4 What is your education level?

□_1_ Primary school or below

□_2_ Junior high

□_3_ Senior high or equivalent

□_4_ College

□_5_ University

□_6_ Postgraduate

3-5 What is your monthly income level?

□_1_ Below 1000RMB

□_2_ 1000-2999 RMB

□_3_ 3000-4999 RMB

□_4_ 5000-6999 RMB

□_5_ 7000-9999 RMB

□_6_ 10,000 RMB or above

□_7_ No fixed income

3-8 Are you a frontline worker or a management staff?

□_1_ Frontline worker □_2_ Management staff

**End of Questionnaire**
